# Unveiling the hidden: identification and management of overlooked blood vessels in laparoscopic left hemicolectomy for splenic flexure cancer

**DOI:** 10.1186/s12893-024-02424-0

**Published:** 2024-04-27

**Authors:** Wenjun Luo, Peng Chen, Qiang Du, Lie Yang, Zongguang Zhou

**Affiliations:** 1 Division of Gastrointestinal Surgery, Department of General Surgery, West China Hospital of Sichuan University, No. 37 Guoxue Lane, Chengdu, Sichuan Province 610041 China; 2 Institute of Digestive Surgery, State Key Laboratory of Biotherapy and Cancer Center, West China Hospital, Sichuan University, Chengdu, Sichuan 610041 China

**Keywords:** Overlooked blood vessels, Laparoscopic left hemicolectomy, Splenic flexure cancer

## Abstract

**Background:**

During laparoscopic left hemicolectomy procedures, a previously overlooked consistently thick blood vessel within the gastrocolic ligament near the splenic hilum may contribute to post-operative bleeding complications. The purpose of this study was to investigate the identification and management of the previously overlooked blood vessel.

**Methods:**

This is a retrospective descriptive study of patients undergoing laparoscopic left colectomy for splenic fexure cancer conducted at a national gastrointestinal surgery centre in China. Consecutive patients with splenic fexure cancer who underwent laparoscopic left colectomy using our“five-step process”(*n* = 34) between January 2021 and July 2023 were included.

**Results:**

The vessels can be effectively exposed using the aforementioned “five-step process.” It was observed that the overlooked vessels consistently present in all patients were identified as the omental branch of the left gastroepiploic artery and vein.

**Conclusion:**

We have identified the origin of previously overlooked blood vessels and recommended a safe method for their management. This may offer advantages to colorectal surgeons performing laparoscopic left colectomy for splenic flexure cancer

**Supplementary Information:**

The online version contains supplementary material available at 10.1186/s12893-024-02424-0.

## Background

Splenic flexure cancer is a relatively rare form of colorectal cancer, accounting for only 3–5% of cases [1–3]. Surgical management of this particular region is challenging due to the complex vascular variability and the proximity of vital organs such as the pancreas, spleen, and duodenum. In addition, a significant number of patients require emergency surgery to relieve mechanical obstruction [4]. These factors collectively heighten surgical risk and pose a significant challenge to surgeons [5–7]. . In order to improve the safety and efficacy of surgical interventions, numerous studies have been conducted in recent years to investigate vascular variants and surgical approaches [8–11]. The results of these studies have empowered surgeons to meticulously dissect and ligate the branching vessels of the mesentery during surgery, thereby aiding in the reduction of postoperative complications [12–14]. .

**Fig. 1:**
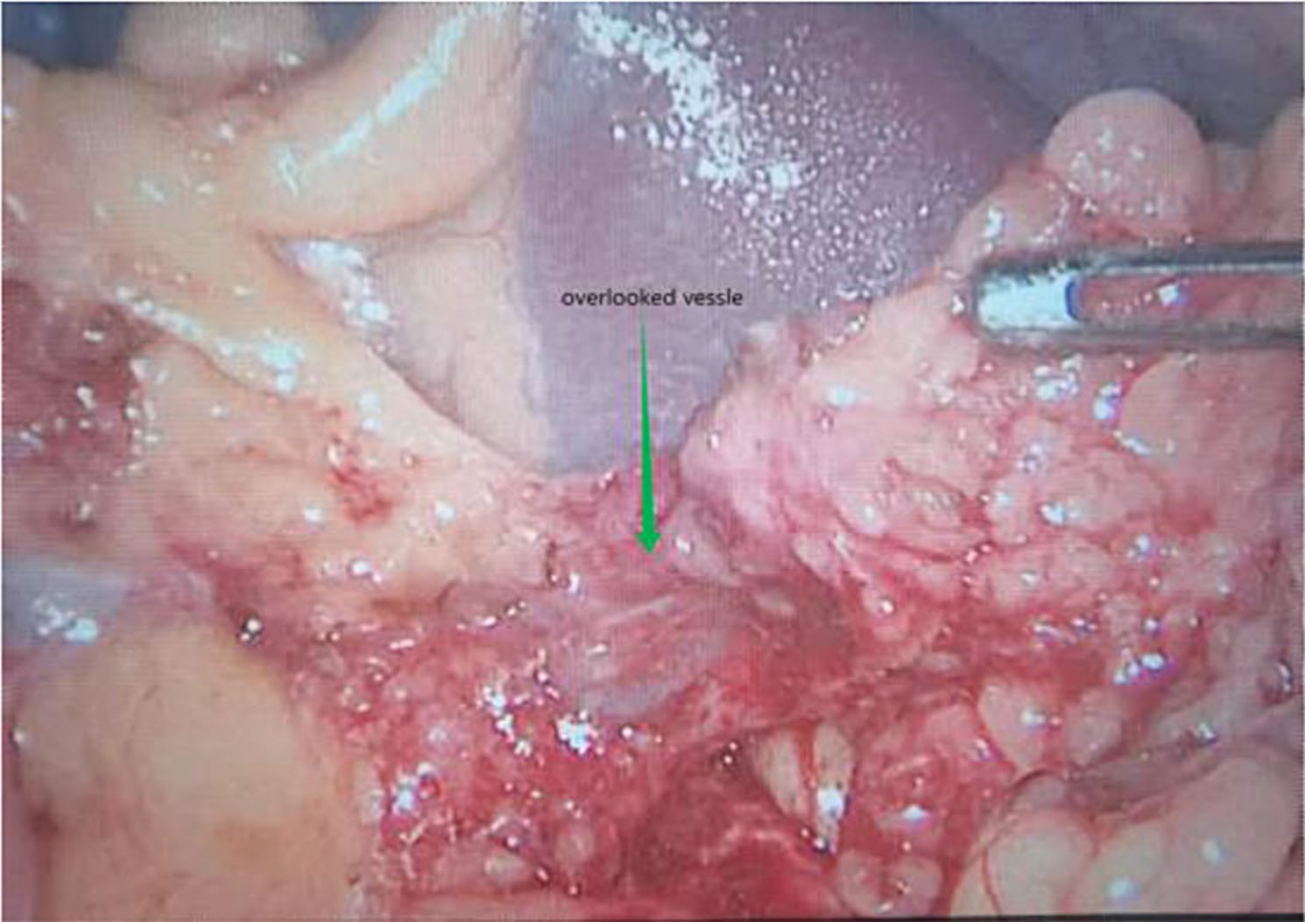
An apparently thickest longitudinal vessel in the gastrocolic ligament near the splenic hilum which we refered as “overlooked vessel” in the context

However, the presence of a seemingly thick and stable longitudinal vessel in the gastrocolic ligament near the splenic hilum, has been regularly overlooked during the resection of the left omentum (Fig. 1) in surgical settings. Currently, comprehensive information regarding the origin, trajectory, and appropriate management techniques of this vessel is lacking. For the sake of convenience, we have informally designated this vessel as the “overlooked vessel” within the scope of this research. The primary objective of this study was to elucidate the origin and pathways of the “overlooked vessel” and propose a safe technique for its management during laparoscopic left colectomy, leveraging our established “five-step process” [15].

## Patients and methods

### Patients

Between January 2021 and July 2023, 34 consecutive patients underwent laparoscopic left colectomy at Department of Gastrointestinal Surgery, West China Hospital were enrolled in this study. The inclusion criteria included patients diagnosed with left colon cancer who underwent a laparoscopic left colectomy. Patients who had previously undergone abdominal surgery, conversion to open surgery; T4b tumor or distant metastasis, or emergent operations were exclued. The study was approved by the ethics committee of West China Hospital.

### Definition of the “overlooked vessels”

We informally defined the “overlooked vessel” as the conspicuously thickest longitudinal vessel located in close proximity to the splenic hilum within the gastrocolic ligament, which would subsequently undergo isolation.

## Procedure

In our department, we conducted laparoscopic left colectomy utilizing the previously reported “five-step process“ [15]. This approach entailed mobilizing the descending colon in a medial to lateral direction, while simultaneously dissecting the root of the transverse mesocolon at the inferior border of the pancreas. Subsequently, access to the omental sac was achieved from a caudal to cephalad direction.

The transection of the gastrocolic ligament commenced from the left half of the transverse colon and extended towards the splenic hilum, where the presence of the “overlooked vessel” was encountered. Meanwhile, the assistant’s right hand exerts a cephalad pull on the gastrosplenic ligament, directing it towards the abdominal wall, while the assistant’s left hand applies a caudal pull on the gastrocolic ligament, also towards the abdominal wall, in order to counteract the tension caused by the “overlooked vessel” and facilitate exposure of the tail of the pancreas and the splenic hilum. Subsequently, the main surgeon proceeded to dissect the vessel’s root at the tail of the pancreas, followed by the dissection of its distal branches, including the gastric branch towards the greater curvature of the stomach and the omental branch towards the omentum.

After ligating the vessel distal to the gastric branch using vascular clips, the omental branch was subsequently isolated.

The surgical procedure is prescribed and presented in the supplementary video.

## Results

### Patient demographics

The patients consisted of 21 men (61.8%) and 13 women (38.2%), ranging in age from 28 to 84 years (median age, 59 years) (Table 1).

**Table 1:** Baseline clinical and pathological characteristics

	No. patient (n = 34)
Age (y)a	59 (28–84)
Gender	
Men	21 (61.8%)
Women	13 (38.2%)
BMI (kg/m2)a	21.6 (18.4–25.8)
ASA	
I	0 ( 0%)
II	19 ( 55.9%)
III	15 (44.1%)
Pathological T stage	
T1	1( 2.9%)
T2	4 ( 11.8%)
T3	11 ( 32.4%)
T4	18(52.9%)
Pathological N stage	
N0	9(26.5%)
N1	11 (32.4%)
N2	14(41.1%)
Pathological M stage	
M0	34 (100%)
M1	0

^a^Data are expressed as a median (range)

Table 2 demonstrates the surgical and pathologic outcomes of laparoscopic CME and central vascular ligation for left colectomy using the “five-step process”. The mean estimated blood loss was 25 mL, the mean operative time was 185 min, and no postoperative bleeding occured.

**Table 2:** Intraoperative and postoperative outcomes

	No. patients (n = 34)
Operative time (min)a	185(160–205)
Intraoperative blood loss (ml)a	25(20–100)
Conversion to open surgery	1
Postoperative hospital stay (day)a	8(5–14)
Major complications	0
Postoperative bleeding	0
Anastomotic leakage/bleeding	0/0
Mortality	0

^a^Data are expressed as a median (range)

### Origination and paths of the “forgotten vessel”

**Fig. 2:**
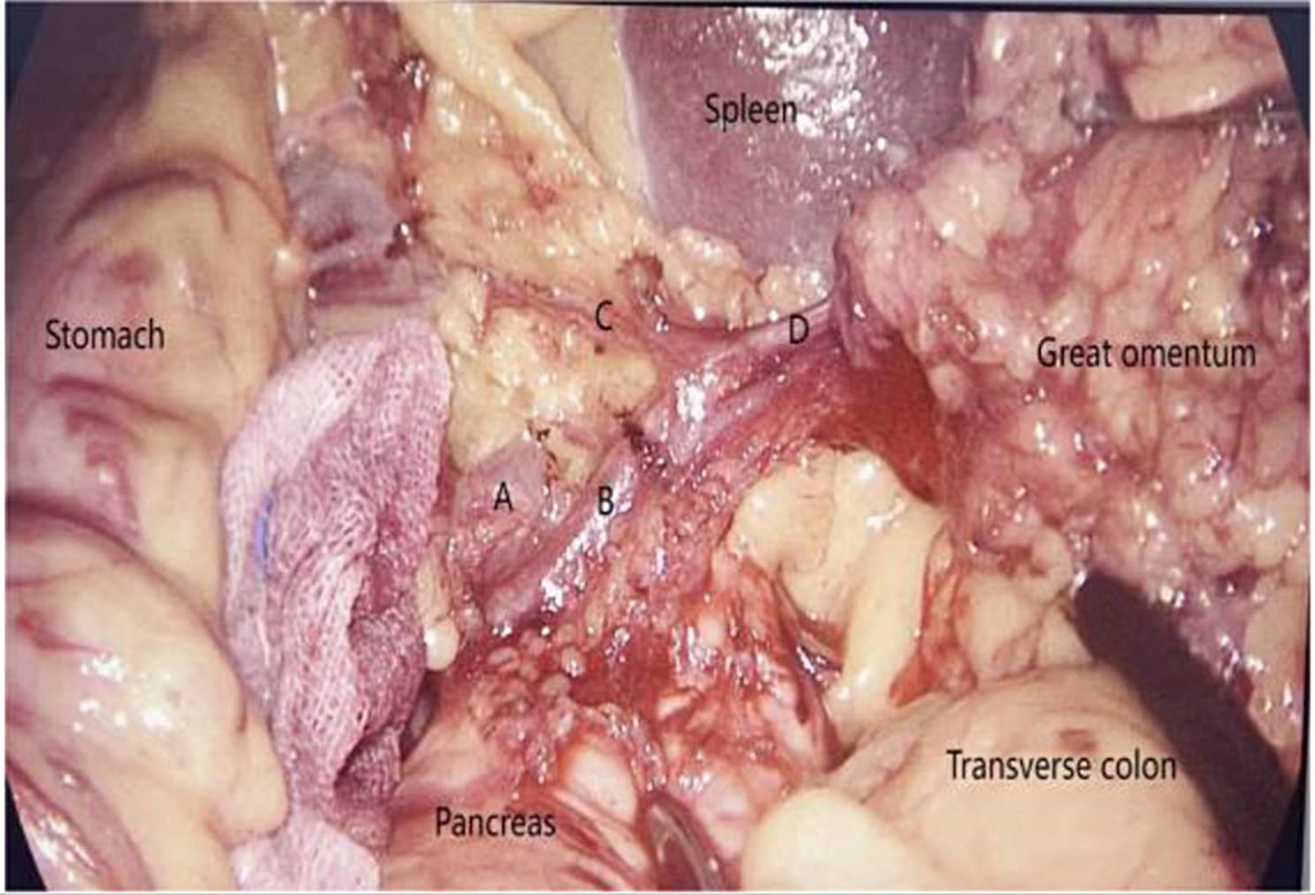
Vascular anatomy of the “overlooked vessel”. **A**: branch of the splenic artery, **B**: left gastroepiploic artery, **C**: gastric branch of the left gastroepiploic artery, **D**: omental branch of the left gastroepiploic artery, i.e. left omentum artery

**Fig. 3:**
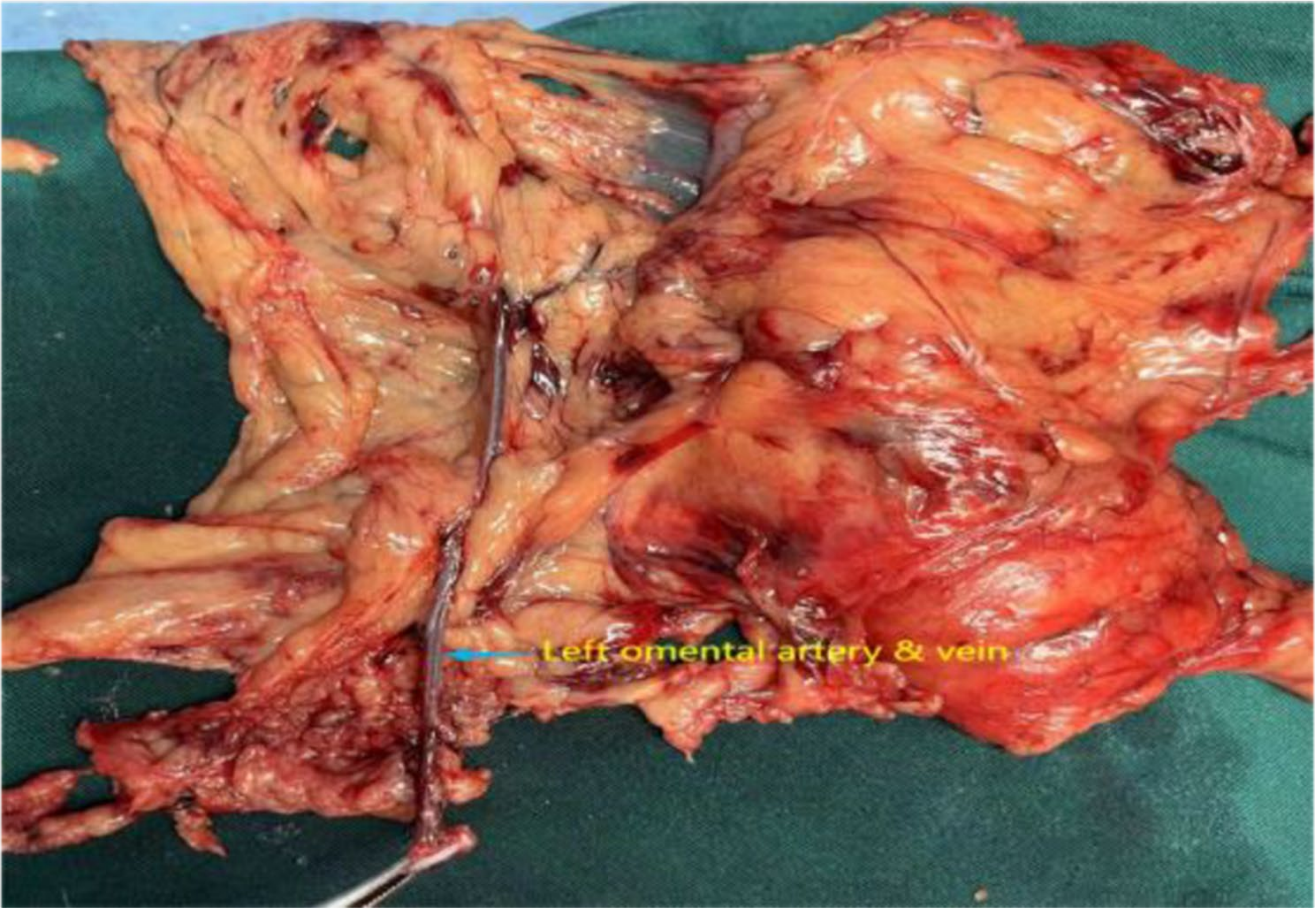
Surgical specimen. The blue arrow indicated the left omental vessels

All 34 patients exhibited the presence of the “overlooked vessel”. Figure 2 illustrated that A represents a branch originating from the splenic artery, while B denoted a vessel that originates from the same branch of the splenic artery and extended towards the stomach and omentum. This observation aligned with the established definition of the left gastroepiploic artery, thus confirming B as the left gastroepiploic artery. The left omentum artery (LOA), also referred to as the “overlooked vessel,” arised from the left gastroepiploic artery and extended towards the greater curvature of the stomach. Its gastric branch was denoted as C, while its omental branch was designated as D. Correspondingly, the accompanying vein is referred to as the left omentum vein. Surgical specimen is shown in Fig. 3.

## Discussion

This study aimed to investigate the origin and trajectory of the “overlooked vessel” during laparoscopic left colectomy in order to enhance procedural safety. The “overlooked vessels” were identified as the omental branch of the left gastroepiploic artery and vein, which we referred to as the left omental artery and vein based on observations made during surgery on living patients. Our findings suggest that the secure management of the vessels during surgery can be achieved by cutting off these vessels after vascular clip ligation. These findings have important implications for surgeons, as they can assist in the safer management of vessels during surgery, ultimately reducing the risk of post-operative bleeding.

The left omental artery, a branch of the left gastroepiploic artery (LGEA), originates from either the splenic artery or its inferior terminal artery. The left omental artery holds significant clinical importance in early gastric cancer surgery as it can be preserved, thus allowing for the preservation of the omentum [16]. Furthermore, in conjunction with the LGEA, the left omental artery is utilized to determine the location of the number 4sb lymph node according to the Japanese Gastric Cancer Association [17]. Conversely, studies focusing on the left omental artery in colon surgery are scarce, potentially due to direct ligation during open surgery. Meanwhile during the laparoscopic left colectomy procedure, the left omental vessels exhibited magnification, resulting in a thicker appearance compared to other vessels within the gastrocolic ligament. Consequently, the origin and nomenclature of this vessel have remained unknown. The persistent uncertainty surrounding this matter prompted our investigation into the source of this vessel. Through a comprehensive analysis of relevant literature, we have determined that the left omental artery (LOA) is an omental branch originating from the left gastric artery (LEGA) [16]. While various variants of the gastric branch of LEGA have been identified, no such variations have been reported for the LOA. This absence of variants may be attributed to the LOA serving as the terminal branch of the LEGA. Furthermore, we refrained from conducting further investigations into the paths of the LOA within the omentum, as its orientation was readily discernible in the surgical specimen. Notably, this study represents the inaugural real-world examination in which the LOA has been identified during laparoscopic left colectomy.

An additional motivation for our exploration of the LOA was to ascertain a secure method of management, given its infrequent mention in colonic surgery and the consequent absence of standardized recommendations for its handling. Due to the increased diameter of this vessel and the potential for heightened postoperative bleeding risk associated with transection using ultrasonic scalpel and other energy platforms, we opted for vascular clip ligation followed by dissection as the preferred approach for managing this vessel. Notably, no instances of postoperative bleeding were observed in this region. Consequently, we propose the utilization of vascular clip ligation followed by cut-off as the recommended method for managing this vessel. Nevertheless, it is imperative to acknowledge that the absence of controlled studies necessitates further investigation to determine the comparative safety of alternative techniques.

As a national gastrointestinal surgery center that performs approximately 100 left colectomies annually, we have amassed extensive expertise in laparoscopic left colectomy. This proficiency has facilitated our study on the “overlooked vessels” in living patients [15]. In this present study, our “five-step process” offers several advantages. Firstly, we initiate dissection at the root of the transverse colonic mesentery, located at the inferior border of the pancreas. This approach ensures that the omental sac has already expanded when the gastrocolic ligament is cut, thereby facilitating the subsequent identification and tracing of the vessels’ origin and pathways. However, caution must be exercised during the transition from the posterior space of the descending colon to the anterior space of the pancreas to prevent inadvertent access to the posterior pancreatic space and potential injury to the splenic vein located posteriorly to the pancreas, which could result in significant hemorrhaging. Secondly, it is recommended to initially disconnect the adhesion between the spleen and the colon to effectively prevent splenic hemorrhage caused by traction.

Previous investigations on vascular aspects have frequently relied on enhanced CT vascular reconstruction or studies conducted on cadavers [18–22].The former method serves the purpose of preoperative assessment of vascular variations and plays a crucial role in facilitating intraoperative vascular dissection. Conversely, the latter method is employed to assess the spatial proximity between blood vessels and nerves or other organs, thereby preventing potential harm to nerves or vital organs during the investigation. Given that the present study does not encompass the aforementioned objectives, these techniques were not employed.

## Conclusion

The presence of the “overlooked vessel” was observed in all patients, identified as the omental branch of the left gastroepiploic artery and vein, which we propose naming the left omental artery and vein. Utilizing only an ultrasonic scalpel for cutting may increase the risk of postoperative bleeding, whereas ligating the vessel with a clip is a safer approach. These findings suggest that surgeons should exercise caution when handling this vessel, as it may aid in preventing postoperative bleeding during laparoscopic left colectomy. A future controlled study will be necessary to investigate safe and effective techniques for managing these vessels.

### Electronic supplementary material

Below is the link to the electronic supplementary material.

Supplementary Material 1

## Data Availability

The datasets used and analyzed during the current study are available from the corresponding author on reasonable request.
